# The WRKY Transcription Factor GmWRKY12 Confers Drought and Salt Tolerance in Soybean

**DOI:** 10.3390/ijms19124087

**Published:** 2018-12-17

**Authors:** Wen-Yan Shi, Yong-Tao Du, Jian Ma, Dong-Hong Min, Long-Guo Jin, Jun Chen, Ming Chen, Yong-Bin Zhou, You-Zhi Ma, Zhao-Shi Xu, Xiao-Hong Zhang

**Affiliations:** 1 College of Life Sciences, College of Agronomy, Northwest A&F University, State Key Laboratory of Crop Stress Biology for Arid Areas, Yangling 712100, China; Shiwy12@126.com (W.-Y.S.); mdh2493@126.com (D.-H.M.); 2 Institute of Crop Science, Chinese Academy of Agricultural Sciences (CAAS), National Key Facility for Crop Gene Resources and Genetic Improvement, Key Laboratory of Biology and Genetic Improvement of Triticeae Crops, Ministry of Agriculture, Beijing 100081, China; duyongtao1994@126.com (Y.-T.D.); jinlongguo@caas.cn (L.-G.J.); chenjun@caas.cn (J.C.); chenming02@caas.cn (M.C.); zhouyongbin@caaas.cn (Y.-B.Z.); mayouzhi@caas.cn (Y.-Z.M.); 3 Faculty of Agronomy, Jilin Agricultural University, Changchun 130118, China; winter0106@163.com

**Keywords:** WRKY, stress responsive mechanism, drought tolerance, salt tolerance, transgenic hairy root assay, soybean

## Abstract

WRKYs are important regulators in plant development and stress responses. However, knowledge of this superfamily in soybean is limited. In this study, we characterized the drought- and salt-induced gene *GmWRKY12* based on RNA-Seq and qRT-PCR. *GmWRKY12*, which is 714 bp in length, encoded 237 amino acids and grouped into WRKY II. The promoter region of *GmWRKY12* included ABER4, MYB, MYC, GT-1, W-box and DPBF *cis*-elements, which possibly participate in abscisic acid (ABA), drought and salt stress responses. *GmWRKY12* was minimally expressed in different tissues under normal conditions but highly expressed under drought and salt treatments. As a nucleus protein, *GmWRKY12* was responsive to drought, salt, ABA and salicylic acid (SA) stresses. Using a transgenic hairy root assay, we further characterized the roles of *GmWRKY12* in abiotic stress tolerance. Compared with control (Williams 82), overexpression of *GmWRKY12* enhanced drought and salt tolerance, increased proline (Pro) content and decreased malondialdehyde (MDA) content under drought and salt treatment in transgenic soybean seedlings. These results may provide a basis to understand the functions of *GmWRKY12* in abiotic stress responses in soybean.

## 1. Introduction

Drought and salinity are the most important abiotic stress factors affecting plants growth and crop yield. On average, 1/3 of cultivable land suffers drought and salinization, which is equivalent to a loss of about 1,500,000 ha of crop land per year [1]. The damage caused by drought and salt are almost the sum of losses caused by other stress factors. Under limited land and water resources, it is necessary to breed new stress-resistant varieties to increase yield and ensure food security. Cultivation of stress-resistant crop varieties is also an important way to ensure high and stable yield of crops. Transgenic technology has become an important way to learn the function of genes in crops [2,3,4].

Being unable to move, plants encounter numerous biotic and abiotic stresses at different developmental stages which include drought, salinity, temperature changes, nutritional deficiency, pathogen invasion and competition from alien species. To overcome these unfavorable conditions, plants have evolved a complex and efficient signaling network, which can produce a series of responses to external stress signals and induce the expression of stress-related genes to protect the normal activities of the cells [5]. Inducible genes encoding proteins can be divided into three categories based on function: the first is functional genes, which are directly involved in stress response and are located downstream in the signaling network, such as HKT [6,7], SALT [8], NHX [9,10], CAX and CHX [11,12,13]. Another is transcription factors (TFs) that regulate the expression of functional genes in the middle of the signaling network, like DREB [14,15], MYB [16], WRKY [17,18], NAC [19,20], bZIP [21,22] and ERF [23,24]. The last group includes a variety of protein kinases, which conduct stress signals and are located upstream of the signaling network, such as GST [25], LEA [26] and FNS [27].

Among the three classes of stress-related genes, the TFs form a connecting link between the beginning and end of the signaling network; WRKYs are among the largest family of plant TFs. The WRKY domain is about 60 residues in length and is named by a conserved WRKY domain, containing the WRKYGQK heptapeptide at the N-terminus followed by a zinc-finger motif CX4-5CX22-23HXH or CX7CX23HXC [28,29]. Based on the number of WRKY domains and the structure of zinc finger motifs, WRKY TFs are divided into three groups. Group I includes two WRKY domains and either a CX4-5CX22-23HXH or CX7CX23HXC zinc-finger motif. Group II WRKY proteins contain a single WRKY domain and a CX4-5CX22-23HXH zinc-finger motif; due to differences in the primary amino acid sequence, Group II can be divided into five subgroups IIa-IIe [29,30]. Group III WRKY proteins have a single WRKY domain and a CX7CX23HXC zinc-finger motif.

As one of the members of the plant TF family, WRKY is heavily studied. Researchers have determined that WRKY TFs participate in various physiological and developmental processes [29], such as seed development [31], seed dormancy and germination [32], senescence [33], development [34], plant immune response [35], pathogen defense [18,36] and insect resistance [37,38]. Recent studies have revealed that WRKY proteins are involved in the signal transduction of plant hormones, like abscisic acid (ABA) [39,40], jasmonic acid (JA) [41] and gibberellin (GA) [39]. Numerous studies have demonstrated that WRKY TFs respond to abiotic stresses [42,43], such as salt [4], drought [44], cold [45] and heat [46,47,48]. There are 74 WRKY TF members in model plant *Arabidopsis* [49] and 18 WRKYs have been suggested to be induced by exposure to salt stress; overexpression of *WRKY25* or *WRKY33* was sufficient to increase *Arabidopsis* NaCl tolerance [50]. Overexpressing *TaWRKY2* and *TaWRKY19* exhibited salt and drought tolerance in transgenic *Arabidopsis* [51]. Moreover, researchers found that *OsWRKY11* directly bound to the promoter of a drought-responsive gene, RAB21, as well as enhanced heat and drought tolerance in transgenic rice seedlings [52,53]. Ectopic expression of *ZmWRKY33* and *ZmWRKY58* in *Oryza* and *Arabidopsis* improved drought and salt tolerance, respectively, in transgenic plants [54,55]. In addition, there is extensive cross-talk between responses to biotic/abiotic stresses and exogenous hormones, for example drought and salt stress with the plant hormones. *Arabidopsis WRKY46*, *WRKY54* and *WRKY70* are involved in Brassinosteroid-mediated drought response and plant growth [43]. Novel cotton WRKY-genes *GhWRKY25* and *GhWRKY6*-*like* confer tolerance to abiotic and biotic stresses in transgenic *Nicotiana* and enhanced salt tolerance by activating the ABA signaling pathway and scavenging reactive oxygen species [56]. SA-inducible poplar *PtrWRKY73* is also involved in disease resistance in *Arabidopsis* [37]. All of these studies illustrated that WRKY TFs play a significant role in plant developmental and physiological processes and abiotic and biotic stresses.

Soybean (*Glycine max*), is an important global cash crop, accounting for 59 percent of the world’s oilseed production (http://soystats.com). Currently, due to its high protein content it is often treated as an important source of protein for both human consumption and as fodder. The demand for soybean is thus increasing rapidly and improving soybean yield has become a major research goal. Soybean productivity is greatly affected by growing environment, such as climatic and soil conditions (drought, salt, metallic pollution and fungus infection). Therefore, it is vital to cultivate soybean varieties that are resistant to stressors.

Recently, many studies based on biotechnological and RNA-Seq approaches have been conducted on soybean WRKY TFs. Researchers have identified 188 soybean WRKY genes genome-wide and 66 of the genes have been shown to respond rapidly and transiently to the imposition of salt stress [30]. In the latest version of the soybean genome (*Wm82.a2v1*), 176 GmWRKY proteins were confirmed and the expression of *GmWRKY47* and *GmWRKY58* decreased upon dehydration, while *GmWRKY92*, *GmWRKY144* and *GmWRKY165* increased under salt treatment [57]. *GmWRKY13* may function in plant growth and abiotic stress. *GmWRKY21* and *GmWRKY54* conferred tolerance to cold stress and salt and drought stress, respectively [58]. Here, based on RNA-Seq and several databases and bioinformatics methods, we identified *GmWRKY12*, which is associated with abiotic stress tolerance by quantitative RT-PCR. Overexpression of *GmWRKY12* could improve tolerance of soybean to drought and salt.

## 2. Results

### 2.1. Identification of GmWRKYs Up-Regulated under Drought/Salt Treatment

The GmWRKYs are distributed in different tissues or located upstream of soybean genes to bind the W-box consensus (TTGACY) in the promoters of target genes, initiating functions such as plant development, pathogen defense, insect resistance, response to biotic and abiotic stress and participating in signal transduction mediated by plant hormones [59,60]. In order to identify the function of genes or to explore whether GmWRKY mRNA expression goes up under biotic and abiotic stress, we conducted RNA-Seq (Tables S5 and S6). RNA-Seq data were used to screen GmWRKYs that are responsive to drought and salt. There were 105 GmWRKYs upregulated after drought treatment and fifty-three GmWRKYs were selected based on the rule that log_2_ (GH_treat/CK1_treat) >1 (Table 1). Nine GmWRKYs were selected from salt treatment RNA-Seq data based on the rule that log_2_ (NaCl_treat/CK2_treat) >1 (Table 2).

### 2.2. Tissue-Specific Expression Patterns of GmWRKYs

**Figure 1:**
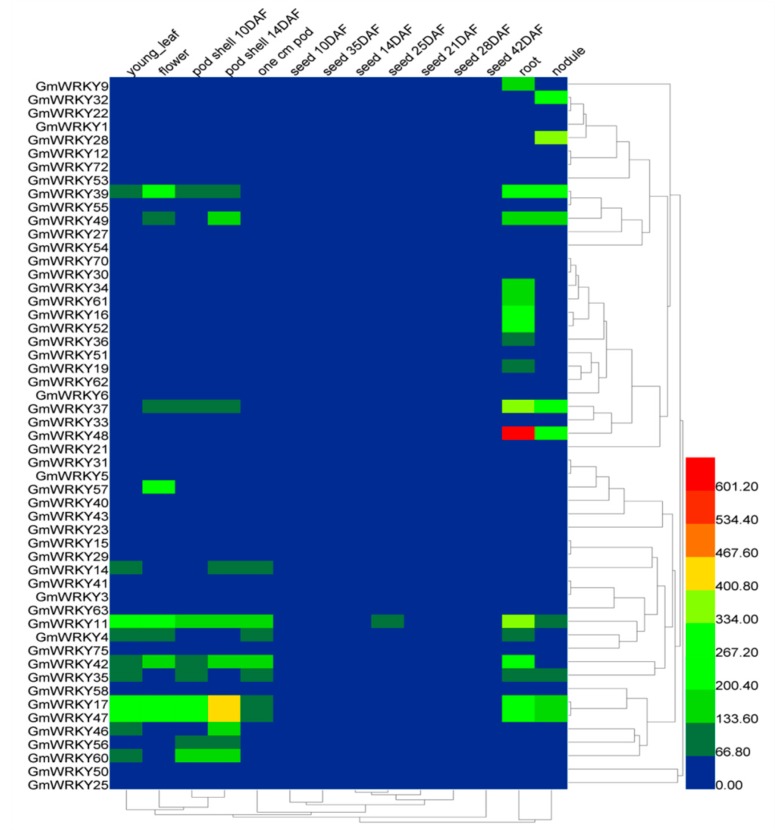
Expression pattern of fifty-three GmWRKYs in six different tissues (young leaf, flower, pod shell, seed, root and nodule). The fifty-three GmWRKYs were selected from drought treatment RNA-Seq data based on the rule that log_2_ (GH_treat/CK1_treat) >1. The tissue expression is from SoyDB (http://soykb.org/). The color legend refers to the different expression level under normal condition. “DAF” in the tissue label indicates days after flowering.

**Figure 2:**
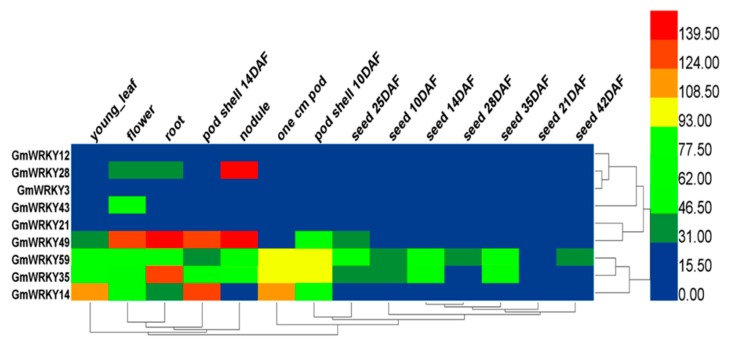
Expression pattern of nine GmWRKYs in six different tissues (young leaf, flower, pod shell, seed, root and nodule). The nine GmWRKYs were selected from salt treatment RNA-Seq data based on the rule that log_2_ (NaCl_treat/CK2_treat) >1. The tissue expression is from SoyDB (http://soykb.org/). The color legend refers to the different expression level under normal condition. “DAF” in the tissue label indicates days after flowering.

To thoroughly study GmWRKY expression profiles under normal conditions, hierarchical clustering was conducted using expression levels of fifty-three (drought-responsive) and nine (salt-responsive) GmWRKY genes in young leaf, flower, one cm pod, pod shell 10 days after flowering (DAF), pod shell 14 DAF, seed 10 DAF, seed 14 DAF, seed 21 DAF, seed 25 DAF, seed 28 DAF, seed 35 DAF, seed 42 DAF, root and nodule (Figure 1 and Figure 2). Approximately 28% of GmWRKYs from different tissues were expressed at low levels or unexpressed (*GmWRKY3*, *5*, *6*, *21*, *22*, *25*, *29*, *30*, *31*, *47*, *50*, *55*, *63*, *70* and *72*); by contrast, 45% of GmWRKYs were highly expressed in different tissues (*GmWRKY4*, *9*, *11*, *14*, *16*, *17*, *19*, *28*, *32*, *34*, *35*, *36*, *37*, *39*, *41*, *42*, *46*, *48*, *49*, *52*, *56*, *57*, *60* and *61*). Among these GmWRKYs, *GmWRKY11* and *GmWRKY17* had the highest expression in four different tissues. *GmWRKY28*, *35*, *37*, *48* and *57* are highly expressed in nodule, seed 10 DAF, seed 42 DAF, root and flower. Within the nine GmWRKYs related to salt response, *GmWRKY3* and *GmWRKY21* had low expression and *GmWRKY14*, *28*, *35*, *49* and *59* were highly expressed in at least four different tissues. The analysis data are available in Tables S1 and S2.

### 2.3. GmWRKYs Responsive to Both Drought and Salt Treatments

**Figure 3:**
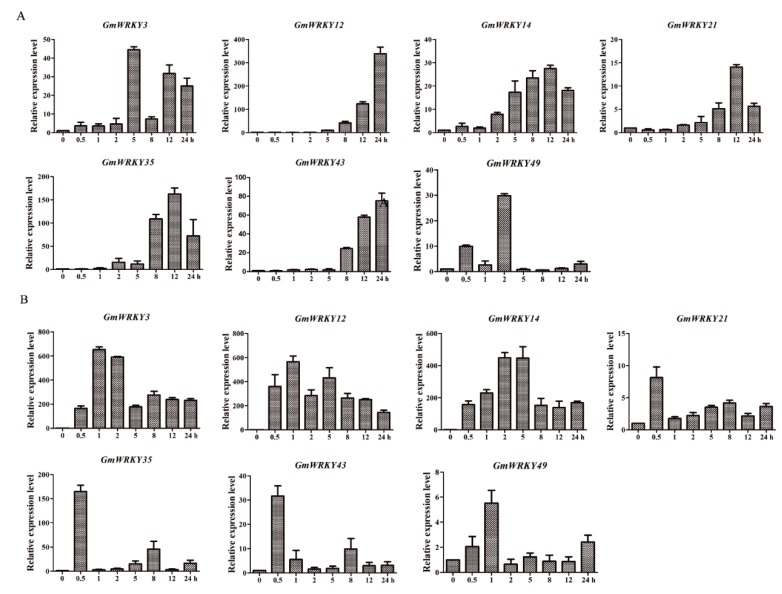
Quantitative RT-PCR of seven GmWRKYs under drought and salt treatment. (**A**) qRT-PCR of seven GmWRKYs under drought treatment. (**B**) qRT-PCR of seven GmWRKYs under salt treatment. The expression level of *GmActin* as a loading control. The data represent means ± SD of three biological replications.

Based on RNA-Seq data and result of Venn method [62], seven *GmWRKY* genes were found to respond to both drought and salt treatments (*GmWRKY3*, *12*, *14*, *21*, *35*, *4*3 and *49*) (Figure S1A). In order to confirm whether the seven GmWRKY genes are responsive to drought and salt, 10-day-old soybean seedlings were subjected to stress treatments. For drought treatment, soybean seedlings were put on filter paper to stimulate drought and then sampled 0.1 g of leaf on different periods (0, 0.5, 1, 2, 5, 8, 12 and 24 h); for salt treatment, the roots of soybean were soaked in 100 mM NaCl solution then sampled 0.1 g of leaf on different periods (0, 0.5, 1, 2, 5, 8, 12 and 24 h), all samples were submerged immediately in liquid nitrogen and stored at −80 °C for RNA extraction then quantitative real-time PCR (qRT-PCR) was conducted. Results confirmed that the seven *GmWRKY* genes were responsive to both treatments (Figure 3). Under drought treatment, the expression levels of *GmWRKY12* and *GmWRKY43* were gradually increased at 0, 0.5, 1, 2, 5, 8, 12 and 24 h. *GmWRKY12* was highly expressed after 12 h of drought treatment. While *GmWRKY14*, *GmWRKY21* and *GmWRKY35* had a tendency to rise first and then decrease, *GmWRKY49* was highly expressed at 2 h. Under drought conditions, the expression profiles of five *GmWRKY* genes were little changed at 0 to 5 h and then increased significantly at 12 h.

Under salt treatment, the expression profile increased first and then decreased, meanwhile, there was a notable change at 0 to 0.5 h and *GmWRKY3*, *12*, *14* and *35* were highly expressed. *GmWRKY12*, which was 714 bp in length, encoded 237 amino acids and had low expression in different tissues under normal conditions but was highly expressed under drought and salt treatments was selected for further investigation (Figure S1B).

### 2.4. Multiple Sequence Alignment and Phylogenetic Analysis of GmWRKY12

**Figure 4:**
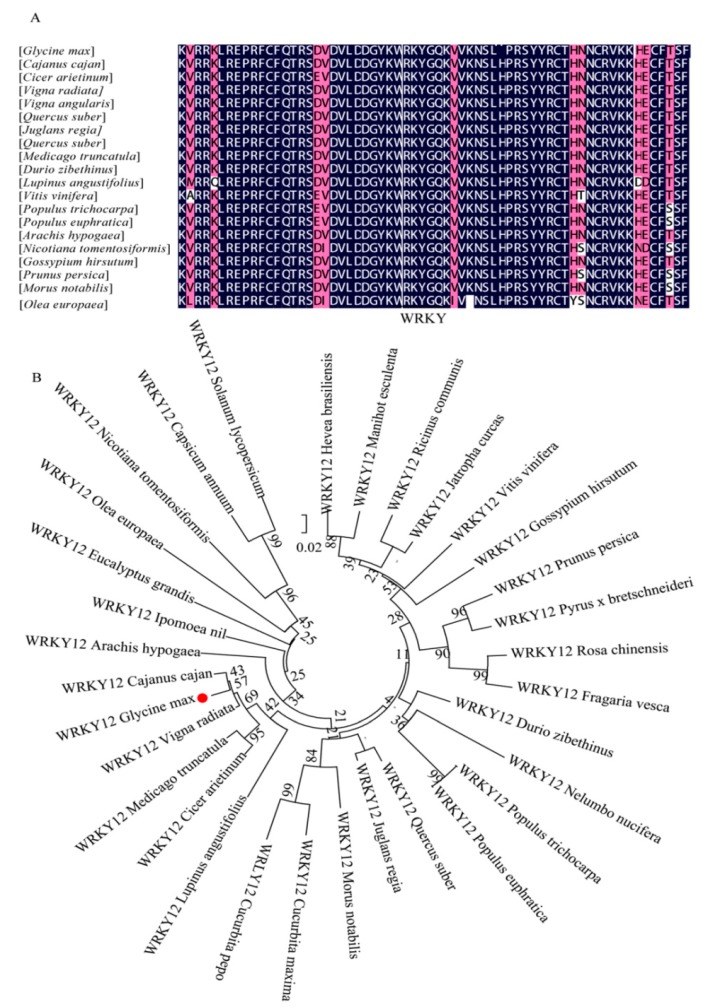
Multiple alignment and phylogenetic relationship of GmWRKY12 with different species. (**A**) Multiple alignment of GmWRKY12 with other WRKY12 proteins from other species. (**B**) Phylogenetic relationship of *GmWRKY12* in different species. The red dot in (**B**) means GmWRKY12. The number of nodes is the bootstrap value and the number on the branch is the evolutionary distance. Bootstrap replications are 1000.

Although WRKYGQK sequence is a conservative motif of WRKY proteins, WRKY variant domains, such as WRKYGEK, WRKYGKK, WQKYGQK, WSKYGQK and WRKYGM have been found in the genomes of *Arabidopsis* [28], rice [63], grape [64] and tomato [65]. This difference may be a variation of WRKY TFs developed over long-term evolution. The domain of these variations is unique and may represent a new type. Therefore, to identify conservation of *GmWRKY12*, *WRKY12* from 20 different species were selected for multiple sequence alignment (Figure 4A). Results showed that 20 species only harbored one WRKY variant WRKYGQK, with amino acid sequence similarity of 75%, which illustrated that *GmWRKY12* was highly conserved. To further evaluate the evolutionary relationship between *GmWRKY12* and *WRKY12* of 32 different species, a phylogenetic tree was constructed with the neighbor-joining method [66]. Phylogenetic results showed that the relationship between *GmWRKY12* and *VrWRKY12* (XP_014515898.1) was the closest (Figure 4B).

### 2.5. Expression Patterns of GmWRKY12 under Different Treatments

**Figure 5:**
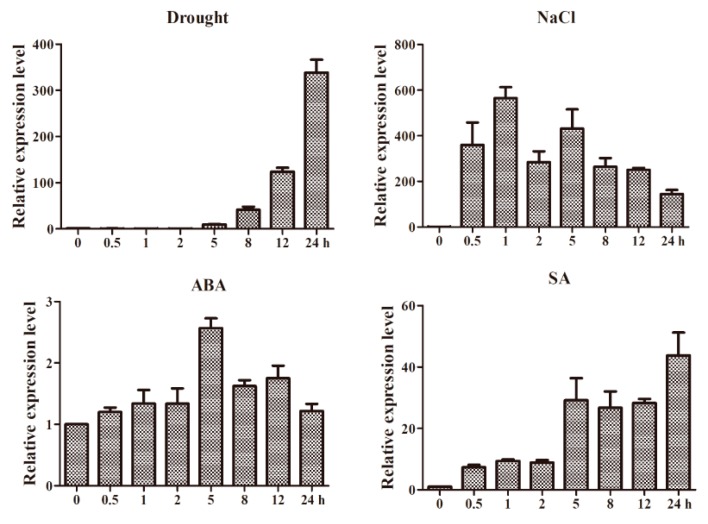
Expression patterns of *GmWRKY12* under drought, NaCl, exogenous ABA and SA. The ordinates are the relative expression level (fold) of *GmWRKY12* compared to non-stressed control. The horizontal ordinate is treatment time for 0, 0.5, 1, 2, 5, 8, 12 and 24 h. The expression level of *GmActin* as a loading control. All experiments were repeated three times. Error bars represent standard deviations (SDs). All data represent the means ± SDs of three independent biological replicates.

*GmWRKY12* was responsive to drought and salt treatments (Figure 3). WRKY proteins are reported to be involved in signal transductions of plant hormones [39]. In order to identify whether *GmWRKY12* was responsive to other abiotic stresses, expression patterns were identified using qRT-PCR. Results indicated that *GmWRKY12* not only participated in drought and salt response but was also responsive to ABA and SA. Under low concentrations of SA, the expression profile of *GmWRKY12* was increased about 50-fold (Figure 5).

### 2.6. Cis-Acting Elements in Promoter

To further understand the regulatory mechanism of *GmWRKY12*, we isolated its promoter region. *Cis*-elements correlated to stress were present in the promoter region, including the ABA and wound responsive elements ABER4 and MYC, drought responsive element MYB, salt stress responsive element GT-1 and wound responsive element W-box. In addition, there was another element that participated in heat and GA response in the promoter region of *GmWRKY12* (Table 3). This analysis suggested that *GmWRKY12* may function in abiotic stress response.

### 2.7. GmWRKY12 was Located in the Nucleus

**Figure 6:**
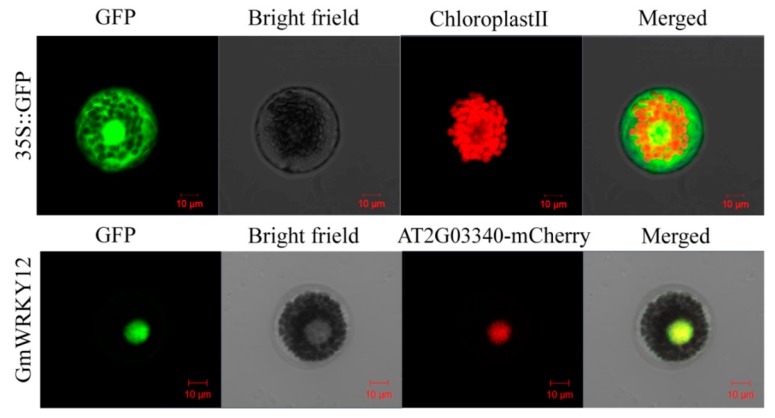
Co-localization of GmWRKY12. The recombinant plasmid of GmWRKY12-GFP and At2G03340-mCherry were co-transformed into wheat mesophyll protoplasts under the control of the CaMV 35S promoter. GmWRKY12 was localized in the nucleus of wheat mesophyll protoplasts protoplasts. Results were observed by a confocal laser scanning microscope (LSM700; CarlZeiss, Oberkochen Germany) after incubating in darkness at 22 °C for 18–20 h. Scale bars = 10 μm.

To investigate GmWRKY12 subcellular localization, GmWRKY12 were fused to the N-terminus of the humanized green fluorescent protein (hGFP) and co-transformed into wheat mesophyll protoplasts with the nucleus marker AT2G03340 (AtWRKY3)-mCherry [67,68]. The 35S::GFP vector was transformed as the control. Fluorescence of GmWRKY12 was specifically detected in the nucleus, whereas GFP fluorescence was distributed throughout the cell (Figure 6).

### 2.8. GmWRKY12 Improved Drought and Salt Tolerance of Soybean

**Figure 7:**
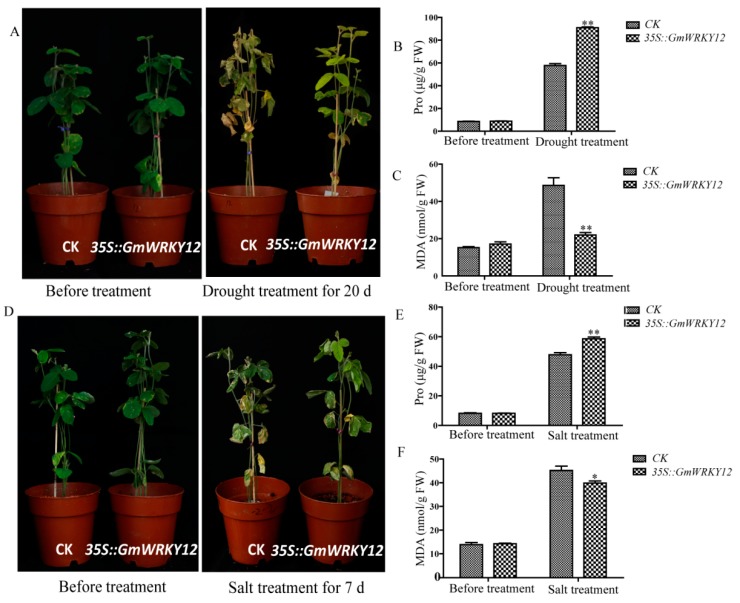
Phenotype identification of *GmWRKY12* under drought and salt treatments. (**A**) Images of drought stress resistance phenotypes of CK and *35S::GmWRKY12* soybean seedlings after drought treatment for 20 days. (**B**) Proline contents in CK and *35S::GmWRKY12* soybean seedlings under normal growth conditions and drought treatment. (**C**) MDA contents in in CK and *35S::GmWRKY12* soybean seedlings under normal growth conditions and drought treatment. (**D**) Images of salt stress resistance phenotypes of CK and *35S::GmWRKY12* soybean seedlings after 200 mM NaCl treatment for 7 days. (**E**) Proline contents in CK and *35S::GmWRKY12* soybean seedlings under normal growth conditions and salt treatment. (**F**) MDA contents in CK and *35S::GmWRKY12* soybean seedlings under normal growth conditions and salt treatment. All data represent the means ± SDs of three independent biological replicates. ANOVA tests demonstrated that there were significant differences (* *p* < 0.05, ** *p* < 0.01).

**Figure 8:**
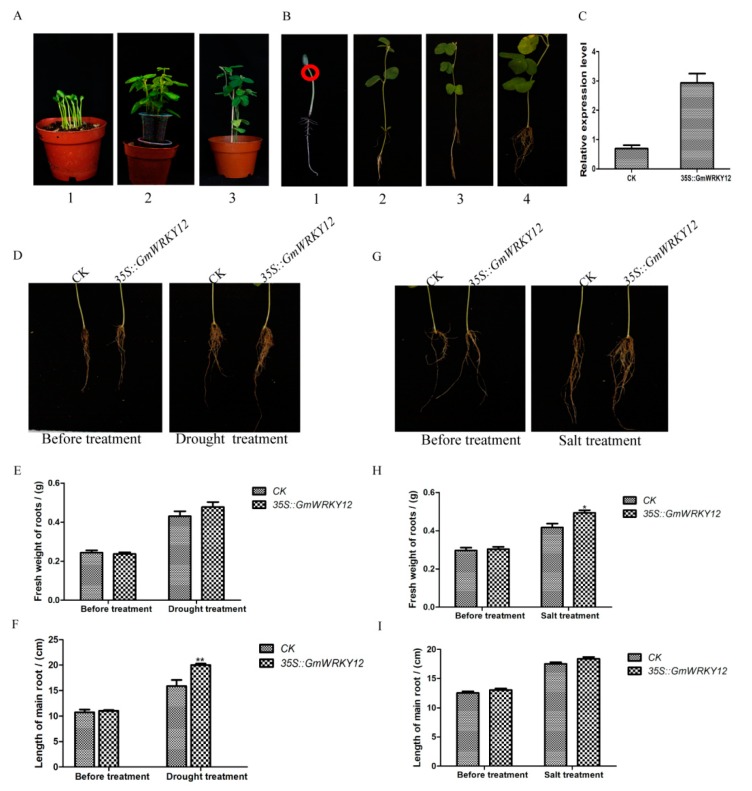
Different growth stage of transgenic soybean seedlings and phenotypes of transgenic soybean hair roots. (**A**) Images of different growth stage of transgenic soybean seedlings cultivated in flowerpot before any treatment. (**A1**) Soybean seedlings of 5-days-old without injected *A. rhizogenes* carrying *GmWRKY12*. (**A2**) Soybean seedlings which have injected *A. rhizogenes* carrying *GmWRKY12* for 7 days. (**A3**) Soybean seedlings which have injected *A. rhizogenes* carrying *GmWRKY12* for 14 days (The original main roots were removed by cutting from 1 cm below the infection site and the hairy roots of the seedlings were cultivated in nutritious soil with full water and grown with 16 h light (100 μM photons m^−2^·s^−1^)/8 h dark at 25 °C). (**B**) Images of different growth stage of signal transgenic soybean seedling before any treatment. (**B1**) Soybean seedling of 5-days-old without injected *A. rhizogenes* carrying *GmWRKY12* and the red circle shows the inject site of *A. rhizogenes.* (**B2**) Soybean seedling which have injected *A. rhizogenes* carrying *GmWRKY12* for 7 days and new hair roots have generated. (B**3**) Soybean seedling which have injected *A. rhizogenes* carrying *GmWRKY12* for 14 days. (B**4**) Soybean seedling that have salt treatment for 7days. (**C**) Relative expression of CK and *35S::GmWRKY12* transgenic soybean hair roots under normal growth conditions. (**D**) Images of drought stress resistance phenotypes of CK and *35S::GmWRKY12* transgenic soybean hair roots after drought treatment for 20 days. (**E**) Fresh weight in CK and *35S::GmWRKY12* transgenic soybean hair roots under normal growth conditions and drought treatment. (**F**) Length in CK and *35S::GmWRKY12* transgenic soybean hair roots under normal growth conditions and drought treatment. (**G**) Images of salt stress resistance phenotypes of CK and *35S::GmWRKY12* transgenic soybean hair roots after 200 mM NaCl treatment for 7 days. (**H**) Fresh weight in CK and *35S::GmWRKY12* transgenic soybean hair roots under normal growth conditions and salt treatment. (**I**) Length in CK and *35S::GmWRKY12* transgenic soybean hair roots under normal growth condition and salt treatment. All data represent the means ± SDs of three independent biological replicates. ANOVA tests demonstrated that there were significant differences (* *p* < 0.05, ** *p* < 0.01).

We further used transgenic hairy root assays to investigate the roles of *GmWRKY12* in abiotic stress responses. Amplified cDNA sequence of *GmWRKY12* was constructed into pCAMBIA3301 to create an overexpression transgenic line and the control was pCAMBIA3301 plant expression vector with CaMV35S promoter. Two constructs were transferred into *Agrobacterium rhizogenes* strain K599 (NCPPB2659) [69] then transformed into soybean hairy roots as previously described [70,71]. After drought treatment for 20 days, both control and over-expression soybean seedlings had leaf shedding to different degrees, especially the old leaves of the plants (Figure 7A). However, compared with transgenic soybean seedlings, the control seedlings were severely wilted and almost 99% of the leaves had serious dehydration and drying. By contrast, there was slight shedding of the old leaves of transgenic soybean seedlings but the new leaves were still growing vigorously. Results of proline and malondialdehyde (MDA) content determination showed that overexpression of *GmWRKY12* increased proline content in transgenic lines, while the MDA content was decreased due to drought stress (Figure 7B,C). Fresh weight and main length of transgenic soybean hair roots under drought treatment were measured (Figure 8E,F), results showed overexpressed *GmWRKY12* in soybean roots enhanced drought tolerance of soybean by increasing the length of transgenic hair roots and the number of transgenic hair roots.

Meanwhile, under NaCl (200 mM) treatment, control and overexpression soybean seedlings had different degrees of leaf shedding (Figure 7D). Compared with the control, transgenic soybean seedlings were slightly wilted and slowly drying out, while the control seedlings were almost dry due to the osmotic stress. Results of Pro and MDA content in transgenic lines (Figure 7E,F) fresh weight and main length of transgenic soybean hair roots (Figure 8H,I) also showed that *GmWRKY12* improved salt tolerance of soybean. These results demonstrated that *GmWRKY12* confers stress tolerance in transgenic hairy roots.

## 3. Discussion

The WRKY transcription factor superfamily, as a recently described member of the TF family, has been studied by many researchers due to its numerous and diverse biological functions. Since the first reports of WRKY TFs [72], research conducted in different species [4,52,57,73,74] has shown that WRKY TFs play significant roles in plant development and stress responses. Recently, many studies of GmWRKY TFs have been based on biotechnological and RNA-Seq approaches [30,57]. However, these studies mainly reported genome-wide annotation of the WRKYs and structure analysis of some genes involved in response to abiotic and biotic stresses. Although these genes have been identified through biochemistry and bioinformatics approaches, knowledge about soybean stress tolerance was limited. In this study, based on qRT-PCR and RNA-Seq data, *GmWRKY12* was selected for investigation of stress tolerance in soybean (Figure S1).

According to classifications in the WRKY family [18,28,75], WRKY12 belongs to Group IIc and contains a single WRKY domain and a CX4-5CX22-23HXH zinc-finger motif. Recent studies have shown that the WRKYGQK heptapeptide, which can specifically recognize and bind to the W-box consensus sequence (TTGACY) in the promoters of target genes, can be replaced by WRKYGKK, WRKYGEK, WKKYEDK, or WKKYCEDK; variations of the WRKYGQK motif might change the DNA binding specificities to downstream target genes [75]. However, multiple sequence alignment results showed that WRKY12 in different species only harbor the same WRKYGQK heptapeptide, demonstrating that WRKY12 protein is evolutionarily conserved and can recognize and bind to downstream target genes (Figure 4A). The result was consistent with the results observed in other species [54,57,65,76,77]. Structural conservation determines functional specificity: in rice, *OsWRKY12* was related to normal plant growth and expression of *OsWRKY12* was low at the seedling stage but increased gradually with growth [78]; similar results were found in specific tissues in our study. *GmWRKY12* has low expression in young leaf, flower, one cm pod, pod shell 10 DAF, seed 10 DAF, seed 14 DAF, seed 21 DAF, seed 25 DAF, seed 28 DAF, seed 35 DAF, seed 42 DAF and root under normal conditions. At the pod shell 14 DAF and nodule stages, the expression levels gradually increase (Table S2), which may be because genes are differentially expressed at different growth stages, or may perform different activities, such as metabolism, nutrient absorption or material transformation. For example, at the nodule stage, plants are primarily vegetative, while at seed 42 DAF, plants are accumulating nutrients [57]. In addition, WRKY12 was related to plant flowering time: *Arabidopsis* plants overexpressing *MlWRKY12* showed early flowering phenotype [79]. WRKY12 and WRKY13 have opposite effects on flowering time in the action of GA [80]. Overexpression of three *Triticum* genes, *TaWRKY12*, *TaWRKY18* and *TaZFP2* induced the expression of some genes related to Pi absorption and transportation, enhancing the abilities of Pi uptake and Pi use efficiency in plants under low-Pi stress conditions [81]. Thus, *GmWRKY12*, like other WRKYs, is involved in plant growth and development.

There are many *cis*-acting elements in the *GmWRKY12* promoter region, such as MYC (ABA and wound responsive element), W-box (SA responsive element), ABER4 (ABA responsive element), MYB (drought responsive element), CCAATB (heat-responsive element), GT-1 (salt stress responsive element), DPBF (dehydration-responsive element) and GARE (GA-responsive element) (Table 3). The presence of these elements indicates that *GmWRKY12* may take part in various biotic and abiotic responses except for growth and development of plants. Research of tobacco transcription factors *NtWRKY12* and *TGA2.2* found that *NtWRKY12* alone was able to induce PR-1a::GUS expression to high levels, the PR-1a gene was salicylic acid-inducible to activate the expression of SA-inducible genes [82]. SA is an important endogenous molecule that activates plant hypersensitive response and systemic acquired resistance, which are often involved in disease resistance of plants [83]. As the closest orthologue of *AtWRKY12*, *BrWRKY1*2 from Chinese cabbage conferred enhanced resistance to *Pectobacterium carotovorum ssp. carotovorum* (*Pcc*) through transcriptional activation of defense-related genes [84]. Furthermore, *LrWRKY12* were responsive to SA and methyl jasmonate (MeJA) treatments and conferred more resistance to *B. cinerea* than in wild-type plants [85]. These results show that WRKY12 plays an important role in disease defense of plants, mainly because WRKYGQK specifically binds to the W-box to induce expression of downstream target genes.

In addition to the significant roles of WRKY12 identified in development and disease defense of plants, WRKY12 also functions in plant stress responses. Under treatment with NaCl and PEG, the expression level of *THWRKY12* in *Tamarix* tissues was increased, the expression pattern of *THWRKY12* after ABA treatment was approximately the same as the expression level changes under NaCl and PEG treatment, showing that the gene may participate in regulating salt and drought tolerance through the signaling pathway regulated by ABA [86]. In our study, *GmWRKY12* was first screened following both drought and salt treatment using RNA-Seq. In order to confirm whether it was responsive to salt and drought stress, qRT-PCR was conducted and further showed that *GmWRKY12* was highly expressed under drought and salt treatment, which indicated that the gene was related to drought and salt tolerance (Figure 3). *Cis*-acting elements and expression pattern analysis of *GmWRKY12* also showed that it may participate in the ABA signaling pathway (Table 3 and Figure 5). However, compared to the high expression level under drought and salt treatment, on the condition of ABA, *GmWRKY12* had low expression. Resistance identification of *GmWRKY12* using a soybean hairy root assay further showed that *GmWRKY12* may be involved in regulating salt and drought tolerance by promoting the combination of *cis*-acting elements with drought and salt-related genes, thereby enhancing plant resistance (Figure 7). Similar results were also found in other studies [87,88,89].

## 4. Materials and Methods

### 4.1. Identification and Annotation of GmWRKYs Response to Drought/Salt Stress

Identification of the response of GmWRKYs to drought/salt stress was based on RNA-seq data collected from a set of drought and salt stress experiments (Tables S5 and S6). Seeds of Williams 82 were cultivated in a 10 × 10 cm flowerpot (vermiculite: nutritious soil is 1:3), fresh leaf of 10-day-old soybean seedlings were used for RNA-Seq.CK1_treat-Expression represented two independent replicates of plants sampled before any treatment; GH_treat-Expression related to drought treatment for 5 h (put on the filter paper to simulate drought) of soybean plants at room temperature; CK2_treat-Expression without NaCl treatment; and NaCl_treat-Expression salt treatment that soaking soybean roots with 100 mM NaCl solution for 1 h and then sampled for RNA-seq [57,68]. Both log_2_ (GH_treat/CK1_treat) >1, log_2_ (NaCl_treat/CK2_treat) >1 and up-regulated were treated as the rule to select GmWRKYs responding to drought/salt stress. Several databases: NCBI (https://www.ncbi.nlm.nih.gov/pubmed), PlantTFDB (http://planttfdb.cbi.pku.edu.cn/), Phytozome (https://phytozome.jgi.doe.gov/pz/portal.html) and SoyDB (http://soykb.org/), were used to annotate Gene ID, Name, Chromosomal localization, CDS, Protein and Group.

### 4.2. Tissue-Specific Expression Patterns of GmWRKYs

Data of six different tissues (young leaf, flower, pod shell, seed, root and nodule) from different growth periods was available from SoyBase (https://www.soybase.org/soyseq/). Heml1.0 software (http://www.patrick-wied.at/static/heatmapjs/) was used to perform hierarchical clustering of fifty-three and nine GmWRKYs under normal conditions. The analysis data are available in Tables S1 and S2.

### 4.3. RNA Extraction and qRT-PCR

Seeds of Williams 82 was cultivated in a 10 × 10 cm flowerpot (vermiculite: nutritious soil is 1:3), fresh leaf tissue of 10-day-old soybean seedlings were used for RNA extraction of different stress treatment. For drought treatment, soybean seedlings were dried on filter paper then sampled 0.1 g of leaf on different periods (0, 0.5, 1, 2, 5, 8, 12 and 24 h), for salt, ABA and SA treatment, the roots of soybean seedlings were soaked in 100 mM NaCl, 100 μmol·L^−1^ ABA and 100 μmol·L^−1^ SA solution, respectively [68]. Then sampled 0.1 g of leaf on different periods (0, 0.5, 1, 2, 5, 8, 12 and 24 h), all samples were submerged immediately in liquid nitrogen and stored at −80 °C for RNA extraction using RNA prep plant kit (TIANGEN, Beijing, China); cDNA was synthesized using a Prime Script First-Strand cDNA Synthesis Kit (TransGen, Beijing, China) following the manufacturer’s instructions. cDNA of treatment for 0 h was used for screen one highly expressed gene from seven GmWRKYs that response to both drought and salt treatment (Figure S1B). qRT-PCR was performed with Super Real PreMix Plus (TransGen, Beijing, China) on an ABI Prism 7500 system (Applied Biosystems, Foster City, CA, USA). Specific primers of *GmWRKY3*, *12*, *14*, *21*, *28*, *35*, *43*, *49 and* soybean actin primers are listed in Table S4. Three biological replicates were used for qRT-PCR analysis. The 2^−∆∆*C*t^ method was used for quantification.

### 4.4. Gene Isolation and Phylogenetic Analysis of GmWRKY12

Venn2.0 (http://bioinfogp.cnb.csic.es/tools/venny/index.html) was used to screen GmWRKYs that respond to both drought and salt treatment, then qRT-PCR was used to find genes highly expressed under stresses. Full-length *GmWRKY12* was amplified by PCR with specific primers from soybean cDNA (*Williams 82*); primers of *GmWRKY12* are available in Table S4. PCR products were cloned into pLB vector (TIANGEN, Beijing, China) and sequenced for further study. The amino acid sequence of WRKY12 in different species were searched for in the NCBI database on account of the amino acid similarity between GmWRKY12 and WRKY12 in different species is more than 50%. DNAMAN was applied for multiple sequence alignment on the basis of the amino acid similarity between GmWRKY12 and WRKY12 in different species is more than 60%. Phylogenetic trees were constructed using MEGA 6.0 with the neighbor-joining method [66] and 1000 bootstrap replications. Information of WRKY12 in different species is listed in Table S3.

### 4.5. Co-Localization of GmWRKY

Seeds of Kenong199 were cultivated in a 10 × 10 cm flowerpot (vermiculite: nutritious soil is 1:3), fresh leaf tissue of 7-day-old wheat seedlings were used for preparation of wheat protoplasts. Amplified cDNA sequence of *GmWRKY12* was cloned into the N-terminus hGFP protein driven by the CaMV35S promoter. The cDNA coding sequences of AT2G03340 (AtWRKY3) which located in the nucleus [67] were fused to the N-terminus of the mCherry protein (WRKY25-RFP) under the control of the CaMV 35S promoter [68]. The recombinant plasmid of GmWRKY12-GFP and AtWRKY3-mCherry were co-transformed into wheat mesophyll protoplasts via the PEG4000-mediated method. The 35S::GFP vector was transformed as the control. Fluorescence was observed using a confocal laser scanning microscope (LSM700; CarlZeiss, Oberkochen, Germany) after incubating in darkness at 22 °C for 18–20 h. Primers are available in Table S4.

### 4.6. Cis-acting Elements in Promoter

The 2.0 kb promoter region upstream of the ATG start codon in the promoter of *GmWRKY12* was obtained from soybean genomic DNA in the Ensembl Plants website, *cis*-acting elements were analyzed by PLACE (http://www.dna.affrc.go.jp/PLACE/).

### 4.7. A. rhizogenes-mediated Drought and Salt Stress Assays

To generate a transgenic line of soybean the amplified cDNA sequence of *GmWRKY12* was constructed into pCAMBIA3301 for an overexpression transgenic line (*35S::GmWRLY12*) and the control was pCAMBIA3301 plant vector with CaMV35S promoter (CK) and two constructs transferred into *A. rhizogenes* strain K599 (NCPPB2659) [69]. Primers are available in Table S4. Williams 82 was cultivated in a 10 × 10 cm flowerpot (vermiculite: nutritious soil is 1:3) for stress experiments (Figure 8A1), soybean seeds were grown under a photoperiod of 16 h light (100 μM photons m^−2^·s^−1^)/8 h dark at 25 °C. When plants displayed two cotyledons (Figure 8A1), *A. rhizogenes* strain K599 harboring pCAMBIA3301 (CK) and K599 harboring *35S::GmWRLY12* were injected at the cotyledonary node and/or hypocotyl (Figure 8B1). A plastic cup was used to surround the inoculated soybean seedlings to provide high humidity conditions. After 3 days, nutritious soil was prepared and used to fill the gaps in the plastic cup so that soybean seedlings could grow new roots (Figure 8A2); plants typically need two weeks to generate new roots (2–10 cm) at the infection site (Figure 8B2,B3). The original main roots were removed by cutting from 1 cm below the infection site and the hairy roots of the seedlings were cultivated in nutritious soil with full water and grown with 16 h light (100 μM photons m^−2^·s^−1^)/8 h dark at 25 °C for 5 days [70,71]. Each flowerpot cultivated 5 transgenic soybean seedlings and 5 replications of each stress treatment (Figure 8A3). Afterward, the transgenic soybean seedlings were subjected to natural dehydration and 200 mM NaCl for drought and salt stress assays [19,68]. For drought stress assay, both CK and transgenic soybean seedlings were grown without water for 20 days. For salt stress assay, CK and transgenic soybean seedlings were treated with 200 mM NaCl solution for 7 days. There are some Supplement Materials need to prepare for culturing *A. rhizogenes* strain K599 that harbored (*35S::GmWRLY12*) and the control (CK)., eg: Solidified LB medium with streptomycin sulfate (100 mg/L) and Kanamycin solution(100 mg/L) (10 g tryptone, 5 g yeast extract, 10 g NaCl, 15 g agar per liter), Liquid LB medium containing streptomycin sulfate (100 mg/L) and Kanamycin solution (100 mg/L) [69].

### 4.8. Measurements of Proline and MDA Contents

Both proline and MDA content were measured with the Pro and MDA assay kit (Comin, Beijing, China) based on the manufacturer’s protocols; all measurements were from three biological replicates.

### 4.9. Measurements of Fresh Weight and Main Length

Transgenic soybean hair roots were used to measure the fresh weight and main length. All data represent the means ± SDs of three independent biological replicates.

## 5. Conclusions

In this study, using RNA-Seq, we identified 62 *GmWRKY* genes in the soybean genome that were differently expressed in six different tissues under normal condition. Seven GmWRKYs responded to both drought and salt treatment. Based on the qRT-PCR, GmWRKY12, a nucleus protein of 237 amino acids, belonging to WRKY Group II was identified. It was responsive to salt, drought and exogenous hormones ABA and SA. Results of Agrobacterium rhizogenes-mediated hairy roots assay showed that overexpressing *GmWRKY12* may improve tolerance to drought and salt in soybean. These results provided new insight into the roles of soybean WRKY genes in abiotic stress responses.

## Figures and Tables

**Table 1:** Annotation of *Glycine max* WRKY transcription factors responding to drought stress (up-regulation).

Gene ID ^a^	Name ^b^	Chr	CDS (bp)	Protein (aa)	Group ^c^
GLYMA_14G103100	*GmWRKY40*	14	849	282	IIb
GLYMA_18G056600	*GmWRKY62*	18	1689	542	IIb
GLYMA_17G042300	*GmWRKY6*	17	1173	390	IIe
GLYMA_04G054200	*GmWRKY50*	4	486	161	IIe
GLYMA_01G222300	*GmWRKY22*	1	738	245	IIc
GLYMA_02G293400	*GmWRKY31*	2	1278	425	IIa
GLYMA_04G218700	*GmWRKY21*	4	591	196	I
GLYMA_06G147100	*GmWRKY51*	6	591	196	III
GLYMA_01G224800	*GmWRKY12*	1	714	237	IIc
GLYMA_11G163300	*GmWRKY19*	11	1647	548	I
GLYMA_06G061900	*GmWRKY17*	6	885	294	IIb
GLYMA_10G011300	*GmWRKY54*	10	972	323	IIa
GLYMA_04G223300	*GmWRKY58*	4	954	317	III
GLYMA_18G213200	*GmWRKY57*	18	900	299	III
GLYMA_06G125600	*GmWRKY53*	6	1095	364	IIa
GLYMA_19G217800	*GmWRKY23*	19	873	290	IId
GLYMA_09G280200	*GmWRKY33*	9	1632	543	I
GLYMA_03G002300	*GmWRKY70*	3	747	248	IIc
GLYMA_13G310100	*GmWRKY36*	13	1845	614	IIc
GLYMA_14G200200	*GmWRKY49*	14	1728	575	IIc
GLYMA_16G026400	*GmWRKY60*	16	1122	373	IIc
GLYMA_16G0544001	*GmWRKY75*	16	588	195	IIb
GLYMA_04G223200	*GmWRKY55*	4	1020	339	IId
GLYMA_02G232600	*GmWRKY39*	2	1743	580	III
GLYMA_05G0290001	*GmWRKY72*	5	1785	594	I
GLYMA_03G220100	*GmWRKY41*	5	762	253	IIe
GLYMA_08G021900	*GmWRKY46*	8	1080	356	III
GLYMA_15G003300	*GmWRKY27*	15	921	306	IIb
GLYMA_17G097900	*GmWRKY61*	17	1803	600	IIc
GLYMA_01G128100	*GmWRKY5*	1	1527	508	IId
GLYMA_12G212300	*GmWRKY16*	12	792	263	IIc
GLYMA_08G082400	*GmWRKY28*	8	881	293	III
GLYMA_07G227200	*GmWRKY3*	7	1602	533	IIc
GLYMA_03G256700	*GmWRKY43*	66	1089	362	IIe
GLYMA_15G168200	*GmWRKY42*	15	882	293	IIb
GLYMA_13G289400	*GmWRKY52*	13	798	265	IIc
GLYMA_08G011300	*GmWRKY25*	8	444	147	IId
GLYMA_09G061900	*GmWRKY47*	19	1573	296	IIc
GLYMA_17G222300	*GmWRKY30*	4	555	184	IIa
GLYMA_01G053800	*GmWRKY9*	1	1368	455	IIc
GLYMA_08G118200	*GmWRKY48*	7	789	262	IIc
GLYMA_01G056800	*GmWRKY32*	1	894	297	IId
GLYMA_08G218600	*GmWRKY56*	8	942	313	III
GLYMA_07G262700	*GmWRKY34*	7	1554	517	IIb
GLYMA_03G159700	*GmWRKY15*	1	1017	338	I
GLYMA_11G053100	*GmWRKY14*	11	963	320	I
GLYMA_05G096500	*GmWRKY11*	17	1050	334	I
GLYMA_17G222500	*GmWRKY63*	17	849	278	IIa
GLYMA_08G240800	*GmWRKY4*	2	1572	523	I
GLYMA_03G176600	*GmWRKY29*	5	1308	436	IIc
GLYMA_08G325800	*GmWRKY35*	8	1734	577	IIc
GLYMA_10G138300	*GmWRKY1*	14	1449	482	IIb
GLYMA_06G077400	*GmWRKY37*	6	903	300	III

^a^—The annotated GmWRKYs according to NCBI (https://www.ncbi.nlm.nih.gov/pubmed) and. PlantTFDB (http://planttfdb.cbi.pku.edu.cn/); ^b^—The names of GmWRKYs are given according to SoyDB (http://soykb.org/); ^c^—The grouping is according to [30,61].

**Table 2:** Annotation of *Glycine max* WRKY transcription factors responding to salt stress (up-regulation).

Gene ID ^a^	Name ^b^	Chr	CDS (pb)	Protein (aa)	Group ^c^
GLYMA_11G053100	*GmWRKY14*	9	963	320	I
GLYMA_08G325800	*GmWRKY35*	8	1734	577	IIc
GLYMA_04G218700	*GmWRKY21*	10	591	196	I
GLYMA_14G200200	*GmWRKY49*	18	1728	575	IIc
GLYMA_07G227200	*GmWRKY3*	18	1602	533	IIc
GLYMA_02G115200	*GmWRKY28*	8	881	293	III
GLYMA_03G256700	*GmWRKY43*	16	1089	362	III
GLYMA_06G320700	*GmWRKY59*	6	2331	776	IIc
GLYMA_01G224800	*GmWRKY12*	7	714	237	IIc

^a^—The annotated GmWRKYs according to NCBI (https://www.ncbi.nlm.nih.gov/pubmed) and PlantTFDB (http://planttfdb.cbi.pku.edu.cn/). ^b^—The names of GmWRKYs are given according to SoyDB (http://soykb.org/). ^c^—The grouping is according to [30,61].

**Table 3:** *Cis*-elements analysis of *GmWRKY12* promotor.

*Cis*-Elements	Numbers	Target Sequences	Functions
MYC	32	CANNTG	ABA and wound responsive element
W-box	21	TTGAC/TTTGACY/TGACY	SA responsive element
ABER4	18	ACGT	ABA responsive element
MYB	14	C/TAACNA/G	Drought responsive element
CCAATB	10	CCAAT	Heat-responsive element
GT-1	7	GAAAAA	Salt stress responsive element
DPBF	6	ACACNNG	Dehydration-responsive element
GARE	2	TAACAAR	GA-responsive element

“Numbers” corresponds to the number of *cis*-elements of each type present in the promoter.

## Supplementary Materials

Supplementary materials can be found at http://www.mdpi.com/1422-0067/19/12/4087/s1.

## Abbreviations

ABA	abscisic acid
ABRE	ABA-responsive element
JA	jasmonic acid
SA	salicylic acid
MeJA	methyl jasmonate
qRT-PCR	quantitative real-time PCR
Pro	proline
MDA	malondialdehyde
DAF	days after flowering
GFP	green fluorescent protein
