# Supplementary material for: The WRKY Transcription Factor GmWRKY12 Confers Drought and Salt Tolerance in Soybean
Source: Int J Mol Sci. 2018 Dec 17;19(12):4087. doi: 10.3390/ijms19124087 (PMC6320995; doi:10.3390/ijms19124087)
Supplement: Supplementary file 1 [file ijms-19-04087-s001.zip › Supplementary materials/Table S3.docx]

**Table S3 Information of different species for sequence alignment and phylogenetic tree**

| Species | Locus on NCBI |
| --- | --- |
| WRKY12 *[Cajanus cajan]* | [XP_003529027.1](https://www.ncbi.nlm.nih.gov/protein/XP_003529027.1?report=genbank&log$=prottop&blast_rank=1&RID=XCRKEZ9701R) |
| WRKY12 *[Cicer arietinum]* | [XP_020240107.1](https://www.ncbi.nlm.nih.gov/protein/XP_020240107.1?report=genbank&log$=prottop&blast_rank=4&RID=XCRKEZ9701R) |
| WRKY12 *[Vigna radiata]* | [XP_014515898.1](https://www.ncbi.nlm.nih.gov/protein/XP_014515898.1?report=genbank&log$=prottop&blast_rank=6&RID=XCRKEZ9701R) |
| WRKY12 *[Quercus sube]* | [XP_023870287.1](https://www.ncbi.nlm.nih.gov/protein/XP_023870287.1?report=genbank&log$=prottop&blast_rank=8&RID=XCRKEZ9701R) |
| WRKY12 *[Juglans regia]* | \| \|  \| [XP_018854711.1](https://www.ncbi.nlm.nih.gov/protein/XP_018854711.1?report=genbank&log$=prottop&blast_rank=9&RID=XCRKEZ9701R) \| \| --- \| --- \| \| \| --- \| --- \| --- \| |
| WRKY12 *[Medicago truncatula]* | \|  \| [XP_013443872.1](https://www.ncbi.nlm.nih.gov/protein/XP_013443872.1?report=genbank&log$=prottop&blast_rank=10&RID=XCRKEZ9701R) \| \| --- \| --- \| |
| WRKY12 *[Durio zibethinus]* | \|  \| [XP_022759474.1](https://www.ncbi.nlm.nih.gov/protein/XP_022759474.1?report=genbank&log$=prottop&blast_rank=12&RID=XCRKEZ9701R) \| \| --- \| --- \| |
| WRKY12 *[Vitis vinifera]* | [XP_017417970.1](https://www.ncbi.nlm.nih.gov/protein/XP_017417970.1?report=genbank&log$=prottop&blast_rank=14&RID=XCRKEZ9701R) |
| WRKY12 *[Populus trichocarpa]* | [XP_006375168.1](https://www.ncbi.nlm.nih.gov/protein/XP_006375168.1?report=genbank&log$=prottop&blast_rank=21&RID=XCRKEZ9701R) |
| WRKY12 *[Arachis hypogaea]* | \|  \| [XP_025679646.1](https://www.ncbi.nlm.nih.gov/protein/XP_025679646.1?report=genbank&log$=prottop&blast_rank=29&RID=XCRKEZ9701R) \| \| --- \| --- \| |
| WRKY12 *[Nicotiana tomentosiformis]* | \|  \| [XP_009604138.1](https://www.ncbi.nlm.nih.gov/protein/XP_009604138.1?report=genbank&log$=prottop&blast_rank=32&RID=XCRKEZ9701R) \| \| --- \| --- \| |
| WRKY12 *[Gossypium hirsutum]* | [XP_016735116.1](https://www.ncbi.nlm.nih.gov/protein/XP_016735116.1?report=genbank&log$=prottop&blast_rank=33&RID=XCRKEZ9701R) |
| WRKY12 *[Prunus persica]* | [XP_007219496.1](https://www.ncbi.nlm.nih.gov/protein/XP_007219496.1?report=genbank&log$=prottop&blast_rank=44&RID=XCRKEZ9701R) |
| WRKY12 *[Morus notabilis]* | \|  \| [XP_024029796.1](https://www.ncbi.nlm.nih.gov/protein/XP_024029796.1?report=genbank&log$=prottop&blast_rank=36&RID=XCRKEZ9701R) \| \| --- \| --- \| |
| WRKY12 *[Olea europaea]* | \|  \| [XP_022867670.1](https://www.ncbi.nlm.nih.gov/protein/XP_022867670.1?report=genbank&log$=prottop&blast_rank=49&RID=XCRKEZ9701R) \| \| --- \| --- \| |
| WRKY12 *[Hevea brasiliensis]* | [XP_021640962.1](https://www.ncbi.nlm.nih.gov/protein/XP_021640962.1?report=genbank&log$=prottop&blast_rank=50&RID=XCRKEZ9701R) |
| WRKY12 *[Nelumbo nucifera]* | \|  \| [XP_010258736.1](https://www.ncbi.nlm.nih.gov/protein/XP_010258736.1?report=genbank&log$=prottop&blast_rank=56&RID=XCRKEZ9701R) \| \| --- \| --- \| |
| WRKY12*[Cucurbita maxima]* | [XP_022974265.1](https://www.ncbi.nlm.nih.gov/protein/XP_022974265.1?report=genbank&log$=prottop&blast_rank=58&RID=XCRKEZ9701R) |
| WRKY12 *[Eucalyptus grandis]* | \|  \| [XP_010052790.1](https://www.ncbi.nlm.nih.gov/protein/XP_010052790.1?report=genbank&log$=prottop&blast_rank=60&RID=XCRKEZ9701R) \| \| --- \| --- \| |
| WRKY12 *[Ricinus communis]* | \|  \| [XP_015579263.1](https://www.ncbi.nlm.nih.gov/protein/XP_015579263.1?report=genbank&log$=prottop&blast_rank=61&RID=XCRKEZ9701R) \| \| --- \| --- \| |
| WRKY12 *[Manihot esculenta]* | [XP_021612140.1](https://www.ncbi.nlm.nih.gov/protein/XP_021612140.1?report=genbank&log$=prottop&blast_rank=62&RID=XCRKEZ9701R) |
| WRKY12 *[Pyrus x bretschneideri]* | [XP_009373203.1](https://www.ncbi.nlm.nih.gov/protein/XP_009373203.1?report=genbank&log$=prottop&blast_rank=63&RID=XCRKEZ9701R) |
| WRKY12 *[Jatropha curcas]* | \|  \| [XP_012083720.1](https://www.ncbi.nlm.nih.gov/protein/XP_012083720.1?report=genbank&log$=prottop&blast_rank=64&RID=XCRKEZ9701R) \| \| --- \| --- \| |
| WRKY12 *[Capsicum annuum]* | \|  \| [XP_016557619.1](https://www.ncbi.nlm.nih.gov/protein/XP_016557619.1?report=genbank&log$=prottop&blast_rank=67&RID=XCRKEZ9701R) \| \| --- \| --- \| |
| WRKY12 *[Ipomoea nil]* | \|  \| [XP_019188420.1](https://www.ncbi.nlm.nih.gov/protein/XP_019188420.1?report=genbank&log$=prottop&blast_rank=83&RID=XCRKEZ9701R) \| \| --- \| --- \| |
| WRKY12 *[Rosa chinensis]* | [XP_024176373.1](https://www.ncbi.nlm.nih.gov/protein/XP_024176373.1?report=genbank&log$=prottop&blast_rank=87&RID=XCUZAU3X015) |
| WRKY12 *[Fragaria vesca]* | \|  \| [XP_011469209.1](https://www.ncbi.nlm.nih.gov/protein/XP_011469209.1?report=genbank&log$=prottop&blast_rank=97&RID=XCRKEZ9701R) \| \| --- \| --- \| |
| WRKY12 *[Lupinus angustifolius]* | [XP_019449998.1](https://www.ncbi.nlm.nih.gov/protein/XP_019449998.1?report=genbank&log$=prottop&blast_rank=15&RID=XCRKEZ9701R) |
| WRKY12 *[Solanum lycopersicum]* | [XP_004229735.1](https://www.ncbi.nlm.nih.gov/protein/XP_004229735.1?report=genbank&log$=prottop&blast_rank=98&RID=XCRKEZ9701R) |
| WRKY12 *[Glycine max]* | \|  \| [XP_003529027.1](https://www.ncbi.nlm.nih.gov/protein/XP_003529027.1?report=genbank&log$=prottop&blast_rank=1&RID=XCUZAU3X015) \| \| --- \| --- \| |
| WRKY12 *[Momordica charantia]* | [XP_022151361.1](https://www.ncbi.nlm.nih.gov/protein/XP_022151361.1?report=genbank&log$=prottop&blast_rank=38&RID=XCRKEZ9701R) |
| WRKY12 *[Sesamum indicum]* | \|  \| [XP_011101863.1](https://www.ncbi.nlm.nih.gov/protein/XP_011101863.1?report=genbank&log$=prottop&blast_rank=100&RID=XCUZAU3X015) \| \| --- \| --- \| |
