# Supplementary material for: The WRKY Transcription Factor GmWRKY12 Confers Drought and Salt Tolerance in Soybean
Source: Int J Mol Sci. 2018 Dec 17;19(12):4087. doi: 10.3390/ijms19124087 (PMC6320995; doi:10.3390/ijms19124087)
Supplement: Supplementary file 1 [file ijms-19-04087-s001.zip › Supplementary materials/Table S4.docx]

| **Table S4 Primers designed in this paper** | |
| --- | --- |
| qGmWRKY3-F | GCTCAAAGGGTTGTTCTGCG |
| qGmWRKY3-R | TGTTGAACCAGCCAGAGCAT |
| qGmWRKY12-F | CAATACTGCTGTGGCCGCTA |
| qGmWRKY12-R | ATCCAGTGTTCGCACCTGTT |
| qGmWRKY14-F | CTGCCTCATTTTTCTGCTCTTCT |
| qGmWRKY14-R | CCTCATTTTTCTGCTCTTCTTTGA |
| qGmWRKY21-F | TGTGGATAAGTGGGGTGGTTTG |
| qGmWRKY21-R  qGmWRKY35-F  qGmWRKY35-R | GCTGAACCTGATCCCTTGGTT  CTGCAAAAGCAACTGAGCCA  TGGAAACCTTAGGCGAGGAG |
| qGmWRKY43-F | CATCCGGTCCCCACTTCTCT |
| qGmWRKY43-R | ATATTGGTGGTGGTGACGAGG |
| qGmWRKY49-F | GCTACTATGGGTGTGATGCC |
| qGmWRKY49-R | TGATGTTGGAGGAGAACGGT |
| pLB-GmWRKY12F | ACACCAAAACTAGAAGATCCAGC |
| pLB-GmWRKY12R | AGGTGAAACATTCATGTTCCGATG |
| qGmActin-F  qGmActin-R  AT2G03340-mCherry-F  AT2G03340-mCherry-R | ACATTGTTCTTAGTGGTGGCT  CTGTTGGAAGGTGCTGAG  CTGGAGCTATGGCTTCTCCG  TCCCAACCTCCTCGCTATCA |
| GmWRKY12-GFP-F | TATCTCTAGAGGATCCACACCAAAACTAGAAGATCCAGC |
| GmWRKY12-GFP-R | TGCTCACCATGGATCCAGGTGAAACATTCATGTTCCGATG |
| GmWRKY12-3301F | GGACTCTTGACCATG ACACCAAAACTAGAAGATCCAGC |
| GmWRKY12-3301R | ATTCGAGCTGGTCACCAGGTGAAACATTCATGTTCCGATG |
