# Supplementary material for: The WRKY Transcription Factor GmWRKY12 Confers Drought and Salt Tolerance in Soybean
Source: Int J Mol Sci. 2018 Dec 17;19(12):4087. doi: 10.3390/ijms19124087 (PMC6320995; doi:10.3390/ijms19124087)
Supplement: Supplementary file 1 [file ijms-19-04087-s001.zip › legends for supplementary figures and tables.rtf]

Supplementary Table S1; Analysis data of fifty-three GmWRKY genes for heat map.
Supplementary Table S2; Analysis data of nine GmWRKY genes for heat map.
Supplementary Table S3; Information of homologs of GmWRKY12 in different species.
Supplementary Table S4; Primers used in the paper.
Supplementary Table S5; RNA-Seq data of drought treatment in soybean.
Supplementary Table S6; RNA-Seq data of salt treatment in soybean.
Supplementary Figure S1; Selection of GmWRKY12. A: Venn diagrams of GmWRKYs that respond to both drought and salt treatment. B: qRT-PCR of seven GmWRKYs to screen the highly expressed genes. The expression level of GmActin as a loading control. The data represent means ± SD of three biological replications.
Supplementary Figure S2; Entire Multiple alignment of GmWRKY12 with 20 different species.
