# Supplementary figures and images for: The WRKY Transcription Factor GmWRKY12 Confers Drought and Salt Tolerance in Soybean
Source: Int J Mol Sci. 2018 Dec 17;19(12):4087. doi: 10.3390/ijms19124087 (PMC6320995; doi:10.3390/ijms19124087)

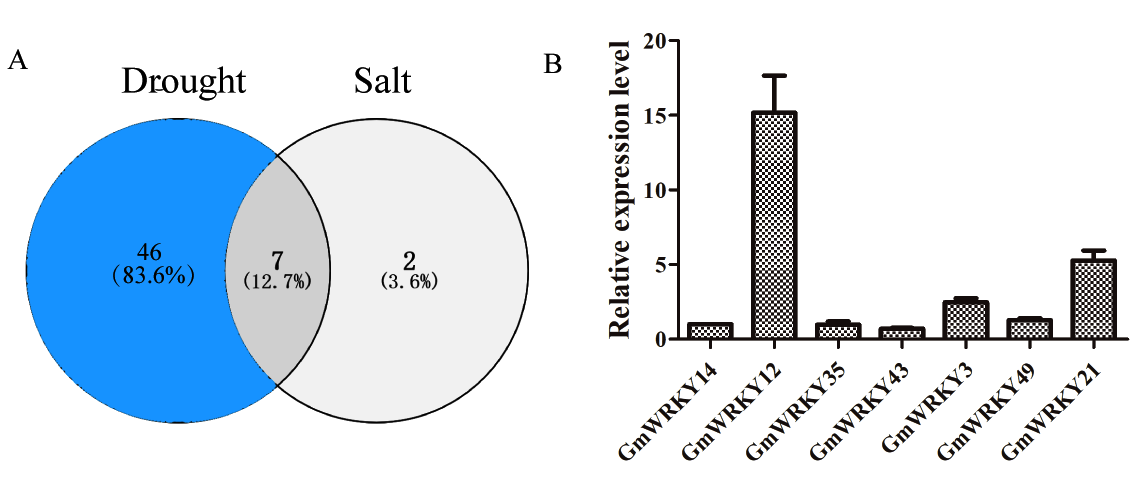

Supplement: Supplementary file 1 [file ijms-19-04087-s001.zip › Supplementary materials/Figure S1.tif]

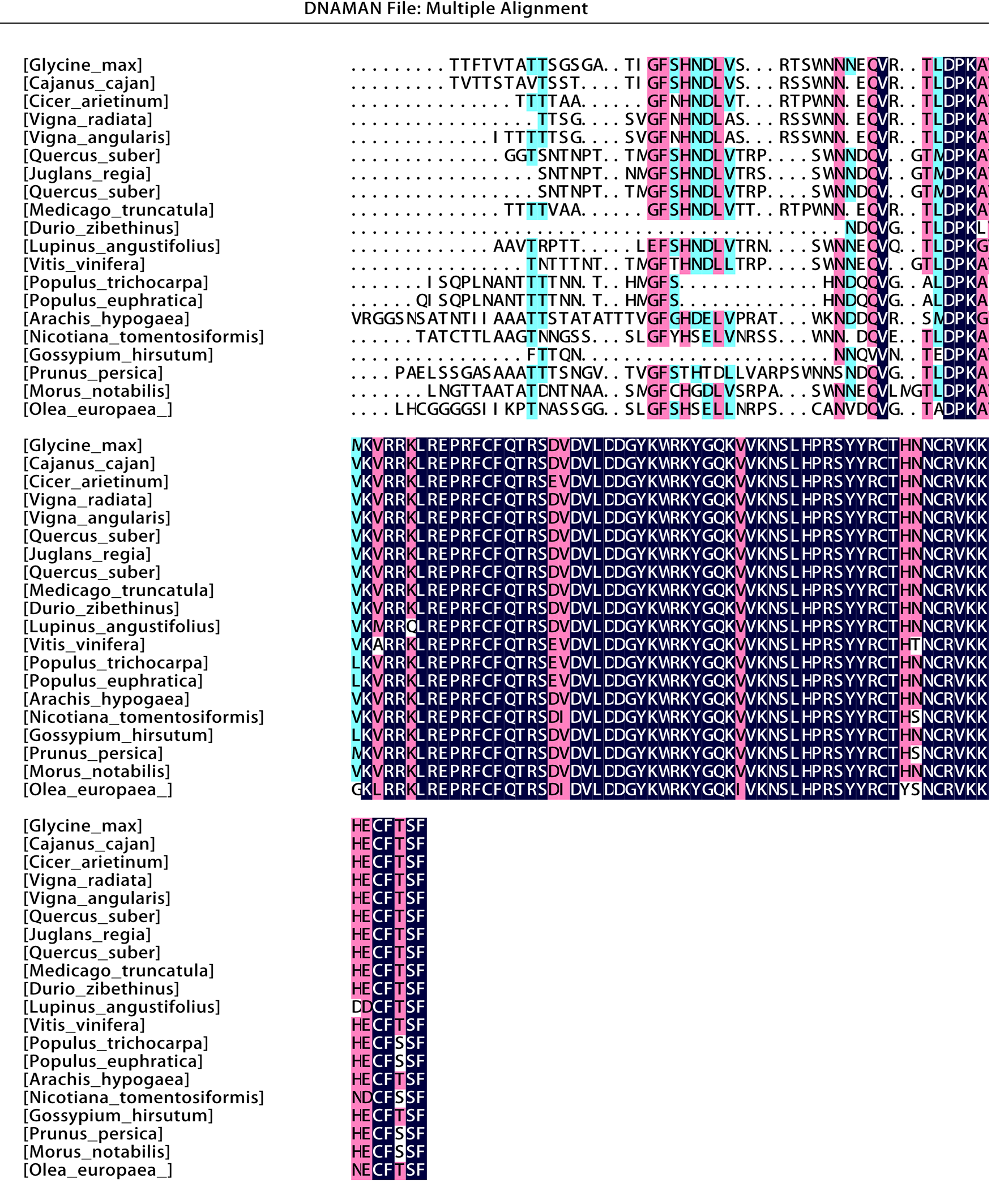

Supplement: Supplementary file 1 [file ijms-19-04087-s001.zip › Supplementary materials/Figure S2.tif]
